# Integrated Collection of Patient-Reported Outcomes and Experiences in Children with Kidney and Hematological Diseases: A Pilot Study

**DOI:** 10.3390/children10071245

**Published:** 2023-07-19

**Authors:** Karolis Ažukaitis, Roma Puronaitė, Goda Elizabeta Vaitkevičienė, Justas Trinkūnas, Augustina Jankauskienė, Danguolė Jankauskienė

**Affiliations:** 1 Clinic of Pediatrics, Faculty of Medicine, Vilnius University, 03101 Vilnius, Lithuania; godaelizabeta.vaitkeviciene@santa.lt (G.E.V.); augustina.jankauskiene@santa.lt (A.J.); 2 Vilnius University Hospital Santaros Klinikos, 08406 Vilnius, Lithuania; roma.puronaite@santa.lt (R.P.); justas.trinkunas@santa.lt (J.T.); 3 Faculty of Fundamental Sciences, Vilnius Gediminas Technical University, 10223 Vilnius, Lithuania; 4 Health Research Laboratory, Mykolas Romeris University, 08303 Vilnius, Lithuania; djank@mruni.eu

**Keywords:** patient-reported outcome, PROM, patient-reported experience, PREM, kidney disease, hematological disease, children, parents

## Abstract

We aimed to explore the feasibility and potential relevance of integrated electronic collection of patient-reported outcome and experience measures (PROM and PREM) in children with special healthcare needs (CSHCN) by using the example of children with kidney and hematological diseases. We performed a cross-sectional, single-center study of children <18 years of age. Children (≥10 years) and their parents received Generic PedsQL Core Scale 4.0 and custom PREM surveys to their email addresses via the REDCap platform, and the results were integrated into the hospital’s electronic health records system. A total of 192 patients (98 with kidney diseases and 94 with hematological diseases) were enrolled. The overall response rate was 51%, and the median time for completion of each proxy questionnaire was approximately three minutes. The lowest PROM scores were observed in the emotional and school functioning dimensions. More favorable experiences in the diagnosis establishment process were associated with higher scores in physical, social, school functioning, and total PROM scores. A better evaluation of the hospital’s environment was associated with higher social functioning, while better information provision correlated with higher physical functioning and total PROM scores. Our data indicates that integrated electronic collection of PROMs and PREMs in the population of CSHCN is feasible, but efforts to increase the response rate are needed. The associations between PROMs and PREMs suggest that future studies exploring targeted interventions at the healthcare service level to improve subjective patient outcomes are needed.

## 1. Introduction

Patient feedback is widely recognized as an invaluable tool for healthcare provision and quality, particularly in the context of the continuous shift towards patient- and family-centered care (PFCC) [1]. Currently, two major instruments are predominantly used to collect patient feedback: patient-reported experience measures (PREMs) and patient-reported outcome measures (PROMs). Although both share the same common denominator—the patient as the sole source of information—the two measures capture qualitatively different information related to health and the healthcare process. PREMs are mainly used to assess what happened during the healthcare delivery process and how patients evaluate it. In contrast, PROMs are used to assess subjective patients health status, usually covering several domains of health, including emotional and social well-being as well as symptoms burden [2,3].

Both PREMs and PROMs allow for insights into different domains of health and healthcare delivery and may serve as useful tools for a number of different purposes. One of those is healthcare quality improvement through different pathways, such as the professional development of healthcare professionals [4] or improved patient-physician interactions and patient satisfaction [5,6], although the impact on individual patient outcomes remains uncertain [7]. Systematic collection and aggregated analysis of PREMs and PROMs targeting specific groups or healthcare sectors may be used for benchmarking, plan-do-study-act (PDSA) models, institution-level analytics, and other purposes [8]. For example, implementation of PREM monitoring has been linked to better safety and clinical efficacy, subjective health outcomes, and patient adherence [9]. On the other hand, whether and how the use of PREMs at macro (healthcare system) or meso (healthcare provider) levels translate into improvements in patient experiences or other health system outcomes at individual levels is yet to be clarified [10].

The American Academy of Pediatrics has recognized psychosocial factors as important contributors to the health and well-being of children with special healthcare needs (CSHCN) and their families [11]. PREMs and PROMs may, therefore, act as important tools for screening and monitoring the psychosocial well-being and experiences of patients and their families. However, although the collection of aggregated PREMs and PROMs tends to be implemented in different healthcare sectors, their use is challenged by feasibility aspects, proper interpretation, and value estimation [8]. Even more challenges may arise in the pediatric population, where different perspectives of children and their parents (or caregivers) may be present, with the additional challenge of being required to answer several questionnaires [12]. Electronic collection of PREMs and PROMs has been shown to be a more attractive option that is also more cost-efficient, may have higher data quality, reach a larger audience, and is less time-consuming than paper administration. On the other hand, the introduction of such systems may be technically challenging, confronted with data protection issues, and may exclude patients who lack computer literacy [13].

In the light of existing evidence and uncertainties, we aimed to explore the feasibility of targeted and integrated electronic collection of PROMs and PREMs in the population of CSHCN by using the example of kidney and hematological diseases. In addition, we aimed to explore the potential implications of such an approach by studying whether using PREMs to capture a broader understanding of experiences throughout the patient journey is associated with patient outcomes at the specific population level.

## 2. Materials and Methods

### 2.1. Study Design and Setting

We performed a cross-sectional study in a single tertiary care center from October 2022 to January 2023. Patients were prospectively enrolled during their visits at the outpatient and daycare clinics. The inclusion criteria were as follows:

1. Patients younger than 18 years of age;
2. Diagnosis of hematological malignancy or severe hematological disease (three or more months after diagnosis) or chronic kidney disorders (children with chronic kidney disease stage 3–5, chronic glomerulopathies, rare kidney diseases, or those on kidney replacement therapy; six or more months after diagnosis).

Patients refusing to participate in the study and non-native speakers were excluded. PREM and PROM screening results and associations between the two measures were assessed as described further, while the feasibility of implementing integrated electronic collection of PREMs and PROMs in the pediatric setting was measured by:

1. Response rate—the ratio of sent and completed questionnaires;
2. Completion time—time from the first answered question to the submission of the questionnaire;
3. Questionnaire completion rate—the proportion of fully completed questionnaires (no missing responses).

Following data protection regulations, all study participants (parents and children ≥10 years old) signed an informed consent form agreeing to provide their email addresses and to receive questionnaire forms. The study was approved by the regional bioethics committee and the hospital’s institutional review board.

### 2.2. Study Organization and Structure

After signing the informed consent form, the parents of the children provided an email address to receive anonymized PREM and PROM questionnaires. All parents received PREM questionnaires irrespective of the age of their child, while PROM questionnaires were only sent to parents of children aged two years or older. In addition, PREM and PROM questionnaires were separately provided to patients who were 10 years of age or older. When available, children received questionnaires to their personal email account; otherwise, a separate email with questionnaires to be completed by children was sent to the parents’ email. Details on questionnaires and their contents are provided further.

All email addresses of parents and their children (when available) were entered into an in-house created quality monitoring module integrated into the hospital’s electronic health records (EHR) system. Study participants then received emails including a unique link to the questionnaire managed via the REDCap (Research Electronic Data Capture) system. Following the completion of the questionnaire, responses were linked back to basic patient data (age of child, disease group: kidney or hematological disease) and were then integrated into PowerBI analytical software within the hospital’s servers for daily-renewing analytics.

### 2.3. PREM and PROM Questionnaires

For the following study, we used custom created PREM questionnaires (for children and parents) and the Generic PedsQL Core Scale (version 4.0, Lithuanian—further referred to as PedsQL) as the PROM tool. All parents of children aged two years and older received PedsQL proxy questionnaires according to the age of the child (13–18, 8–12, 5–7, and 2–4 years), while children who were 10 years or older received PedsQL questionnaires for 13–18 or 8–12 years self-completion. Only one proxy-reported questionnaire was sent per patient with the person completing the questionnaire remaining at the discretion of the family.

PedsQL consists of questionnaires for children and their parents (proxies) that are administered according to the age of the patient, as described previously. Quality of life (QoL) is assessed by calculating an overall QoL score and scoring at four dimensions: physical, emotional, social functioning, and functioning at school/kindergarten. Each of those consists of a 5-point Likert scale question coded from zero to four (from “never” to “almost always”). These answers are then reverse coded to calculate the score and finally converted into a scale from 0 to 100, where 0 indicates the worst possible QoL and 100 the best possible QoL. The subscale scores are calculated by averaging the constituent questions, and the overall PROM score is calculated by averaging the subscale scores. In cases where some questions remain unanswered, the scores are calculated by taking the average of the completed questions, limiting the number of unanswered questions to 50%.

As one of the primary goals was to capture the experiences of children and their parents throughout the whole patient journey in Lithuania, we designed a custom PREM questionnaire. The contents of the PREM questionnaire were based on the results of two previously conducted focus groups that included parents and children (12 participants per group). Focus groups were conducted as unstructured interviews following the principles of the targeted focus group technique [14] in November 2020. All focus group recordings were transcribed verbatim and further underwent content analysis [15] by assigning categories and subcategories using qualitative analysis software Nvivo according to seven categorizing principles [16]. Based on the results of these focus groups, two preliminary PREM questionnaires (for children and parents) were drafted by a study group consisting of healthcare professionals, healthcare management specialists, and policy makers. These primary versions of the PREM surveys were sent for review to representatives of all stakeholders and discussed in an online consensus meeting that included patients, their parents, representatives of patient organizations, healthcare specialists, healthcare managers, and policy makers.

The final PREM surveys consisted of seven questions for children and 12 questions for parents. In addition, two questions concerning time since diagnosis establishment and time since last hospitalization were provided. All PREM questions were coded with a 5-point Likert scale (from 1—completely agree to 5—completely disagree). For the calculation of the total score, the coded responses are recoded into a single scale from 0 to 100, with non-attendance at certain health facilities or non-use of certain services treated as an omitted value. The total PREM score was then calculated by averaging the scores of the questions with a score, allowing for a limit of up to four omitted values. The PREM scores for children were calculated in a similar way. Lower total score values indicate a lower rating of experience, while higher values indicate a higher rating. Examples of the questionnaires (translated into English from Lithuanian) are provided in Table A1 and Table A2.

### 2.4. Psychometric Evaluation

The internal consistency of the questionnaires was evaluated by the Cronbach alpha criterion. Internal consistency was high in all proxy-reported PROM questionnaires (Cronbach alpha > 0.9), except for the 2–4-year-old group (0.631). Similarly, proxy-reported PREM questionnaires showed high internal consistency (Cronbach alpha 0.856), which was similarly lower in the 2–4-year-old group (0.554) but higher than 0.7 in all the remaining groups. Cronbach’s alpha for children reported in PREMs questionnaires was 0.911. Details on internal consistency analysis results are provided in Table A3.

### 2.5. Statistical Analysis

The internal consistency of the questionnaires was assessed by calculating Cronbach’s alpha. Descriptive statistics are presented for the variables and compared between groups (sex, age, etc.) using Pearson’s chi-square, Fisher’s exact criterion, Mann-Whitney U (comparing two groups), or Kruskal-Wallis (comparing more than two groups) criteria for categorical and continuous indicators, respectively. The correlation between responses (within the questionnaire and between total questionnaire scores) was assessed by calculating Spearman’s ρ correlation coefficient.

R software (version 4.2.1) R and its packages REDCapR, PROscorerTools, likert, DescTools, ggplot2, and Hmisc were used for the statistical analysis.

## 3. Results

### 3.1. Characteristics of Studied Population

**Figure 1:**
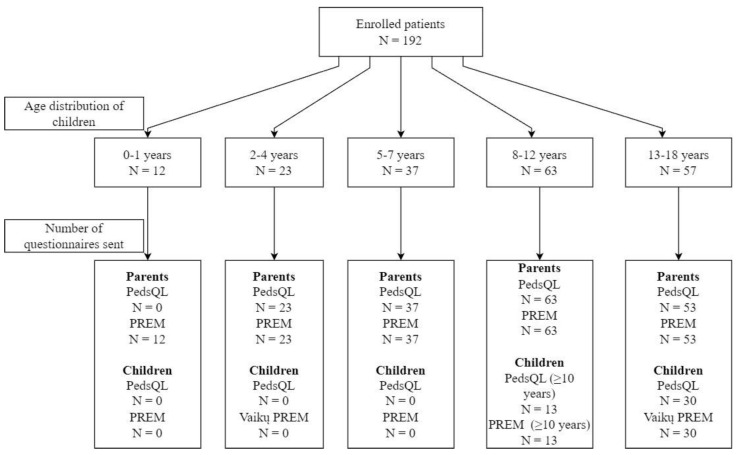
Scheme indicating patient distribution by age groups and the number of different questionnaires sent to parents and children in each group.

In total, 192 patients and their parents were enrolled (98 with kidney and 94 with hematologic diseases), with a median age of 9 years (IQR, 6–13) and 57.8% (*n* = 111) boys. A total of 43 PROM and 43 PREM questionnaires were sent to children, and 176 PROM and 188 (4 email addresses for parents were missing) PREM questionnaires were sent to their parents (Figure 1).

### 3.2. Response and Questionnaire Completion Rates

**Figure 2:**
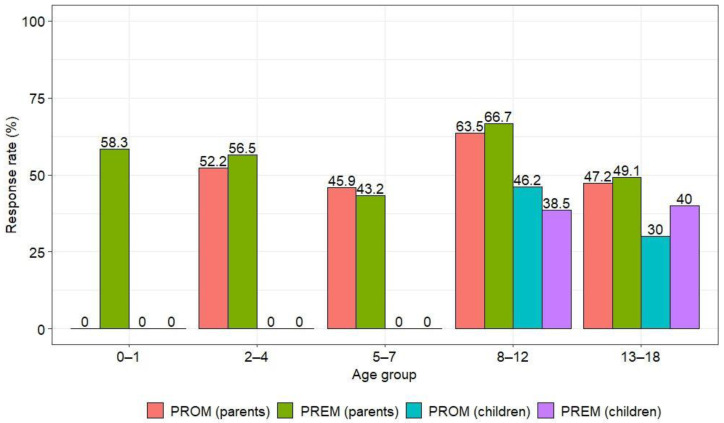
Responses rate by age groups of the children.

The overall response rate was 51.1%. The response rate for PREM questionnaires was slightly higher than for PROM questionnaires (52.4% and 49.8%, respectively), with parents responding more frequently than children (54.4% vs. 37.2%) (Table 1). Similar trends were observed in the kidney and hematological disease groups, but respondents from the kidney disease group responded more frequently than the hematology group (Table A4). The highest number of parental responses was in the group of children aged 8–12 years (Figure 2).

Of the 94 PROMs completed by parents, 84% (*n* = 79) were completed in full (answers provided to all questions), while the remaining PREM questionnaires for parents and PROM and PREM questionnaires for children were fully completed.

### 3.3. Completion Time

The total time to complete both questionnaires by the parents approximated 3 min (median 3.1 and 3.2 min for PROM and PREM, respectively). There was no statistically significant difference in PROM completion time between patient subgroups (kidney and hematology diseases) (*p* = 0.55) and the age of the child (*p* = 0.61), but there was a statistically significant difference between the gender of the child (girls’ parents took longer than boys’ parents, 3.4 vs. 2.9 min, respectively; *p* = 0.03). The time to complete the PREM questionnaire did not differ by primary diagnosis group, age, or gender (all *p* > 0.05).

The median completion time in the children’s group was 2.4 min and 1.5 min for the PROM and PREM questionnaires, respectively, with no differences by age or gender. The data are summarized in Table A5 and Table A6**.**

### 3.4. Results of Patient-Reported Outcomes

**Figure 3:**
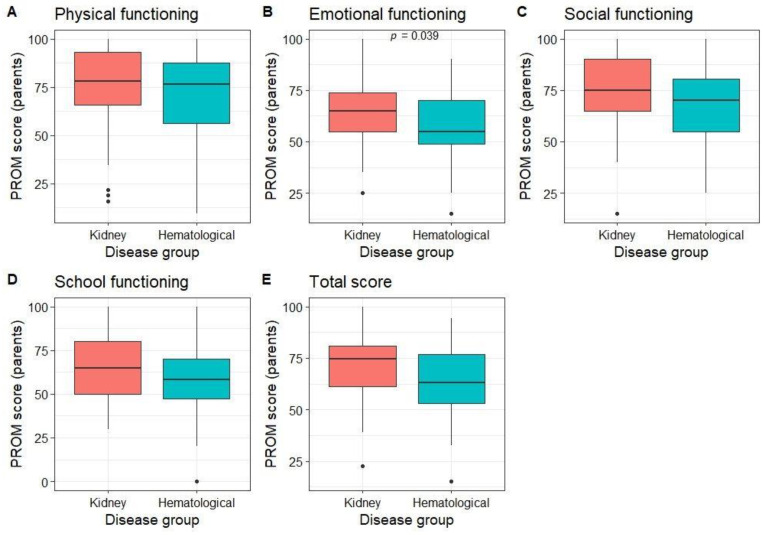
Proxy-reported PROM scores by disease group.

Parents rated their children’s QoL as better than average in all dimensions (median score of 78.1 for physical, 60 for emotional, 75 for social, 60 for functioning at school, and 70 for the total PROM score). Parents of patients with kidney diseases rated their emotional well-being better than those with hematological diseases, with no differences in other dimensions (Figure 3). No differences in PROM scores were observed between different age groups (*p* > 0.05).

**Figure 4:**
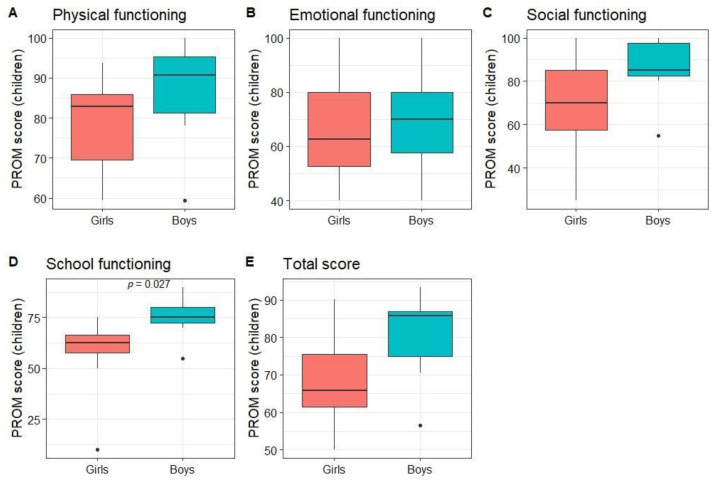
Children-reported PROM scores by gender.

Children rated their own QoL as better than average in all dimensions (median score of 84.4 for physical, 65 for emotional, 85 for social, 70 for school, and 73.9 for total PROM score). When comparing subgroups, no statistically significant differences between patients with kidney and hematological diseases or age groups were found. The only statistically significant difference was between girls’ and boys’ scores on the functioning at school dimension (*p* = 0.03; Figure 4).

### 3.5. Results of Patient-Reported Experiences

Overall, half of the parents (51%) reported that their child’s disease was diagnosed three or more years ago, with a higher proportion in the kidney vs. hematological disease group (69.4% vs. 23.8%). Most of the parents reported that their child has been admitted to the hospital at least once, most frequently less than 1 year ago (47%), with higher rates in the hematological vs. kidney disease groups (69% vs. 32.3%). Data on time since diagnosis and time since last hospitalization by disease group are presented in Table 2.

**Figure 5:**
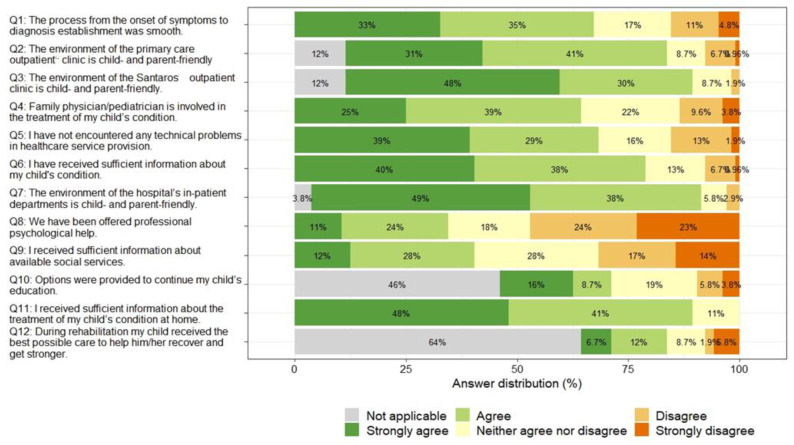
Proxy-reported PREM questionnaire answers by question.

Most parents rated the hospital/department environment and staff behavior positively (Q7, with 87% strongly agreeing or agreeing that the hospital/department environment is friendly for the child and the parents), as well as the provision of information about treatment at home (Q11) and the environment and staff behavior of the children’s outpatient department (Q3). Parents identified the most problematic areas as not being offered psychological support (Q8, with 47% strongly disagreeing or disagreeing that they were offered psychological support) and not being given information about available social services (Q9, with 31% strongly disagreeing or disagreeing that they were given enough information about available social services). A total of 64% of parents indicated that their children did not benefit from rehabilitation services (Q12), and 46% indicated that their children did not have a need for continuing education (Q10). The overall results of the PREM questionnaire for parents are summarized in Figure 5.

Parents rated their overall experience in the health care system more favorably than unfavorably, with an overall PREM score that was higher than the median possible score (median 72.5). PREM scores did not differ by the age or gender of the child, but parents of children with hematological diseases reported an overall more positive experience than those with kidney diseases (median 77.4 vs. 67.5, *p* < 0.001).

**Figure 6:**
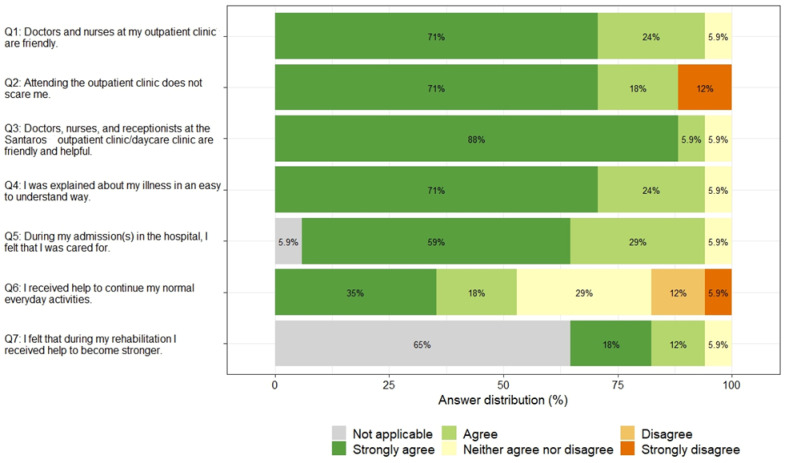
Children-reported PREM questionnaire answers by question.

The majority of children rated the behavior of the staff at the primary care and specialized clinics mostly favorably (Q1 and Q3, 71% and 88% strongly agreed that the staff are friendly and helpful), 12% of children strongly disagreed that attending outpatient clinic does not scare them (Q2), and only 53% of children strongly agreed or agreed that they received help to continue their normal everyday activities. The overall the results of PREM questionnaire for children are summarized in Figure 6.

Children rated their experiences more favorably than unfavorably, with an overall PREM score that was on average quite high (median 89.3), with no statistically significant differences between children with nephrological and onco-haematological diseases, between younger and older children, or between girls and boys (all *p* > 0.05).

### 3.6. Association between Proxy- and Children-Reported Outcomes and Experiences

**Figure 7:**
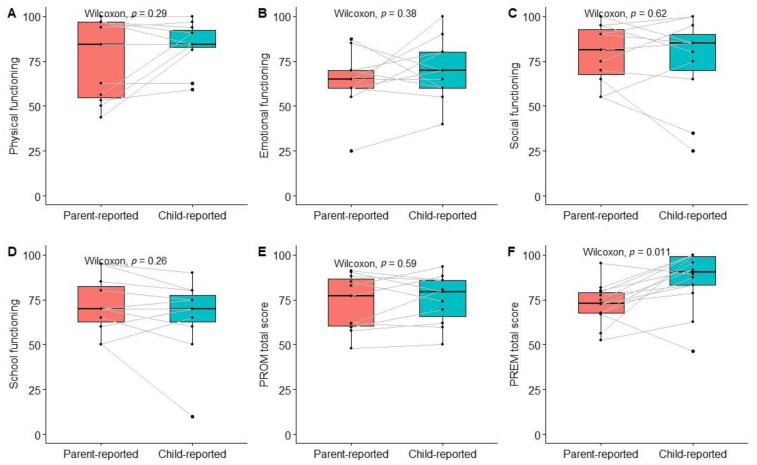
Pair-wise comparison of PROM and PREM scores between children and their parents. (**A**) Physical functioning; (**B**) Emotional functioning; (**C**) Social functioning; (**D**) School functioning; (**E**) PROM total score; (**F**) PREM total score.

The total PROM score and scores for different dimensions did not differ significantly between children and their parents; however, the overall experience score was significantly higher among children compared to their parents (Figure 7).

### 3.7. Association between PROM and PREM Scores

Due to the small sample size of the children’s group, associations between PROM and PREM questionnaire results were only assessed in the parents group. As the coding of answers for individual PROM dimensions (higher score indicating better functioning) and PREM questions (higher score indicating worse experience) was in the opposite direction (except for total scores for PROM and PREM), a negative correlation suggests similar directions for positive/negative outcomes/experiences.

**Figure A1:**
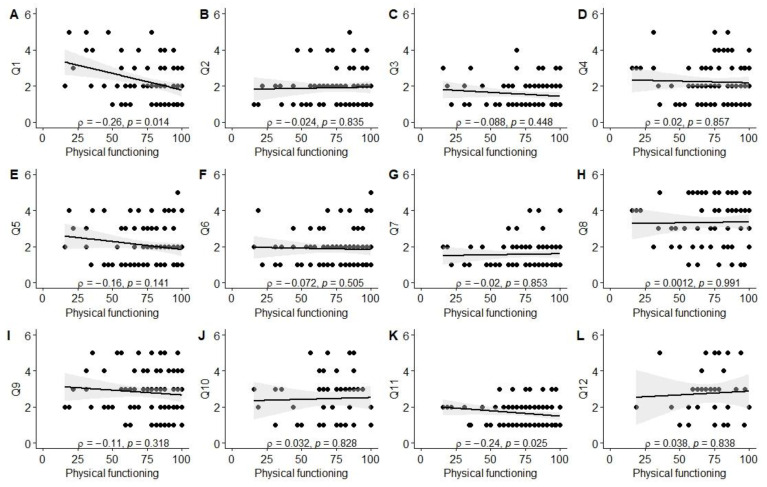
Correlation between physical functioning score and PREM answers. (**A**) PREM question 1; (**B**) PREM question 2; (**C**) PREM question 3; (**D**) PREM question 4; (**E**) PREM question 5; (**F**) PREM question 6; (**G**) PREM question 7; (**H**) PREM question 8; (**I**) PREM question 9; (**J**) PREM question 10; (**K**) PREM question 11; (**L**) PREM question 12.

**Figure A2:**
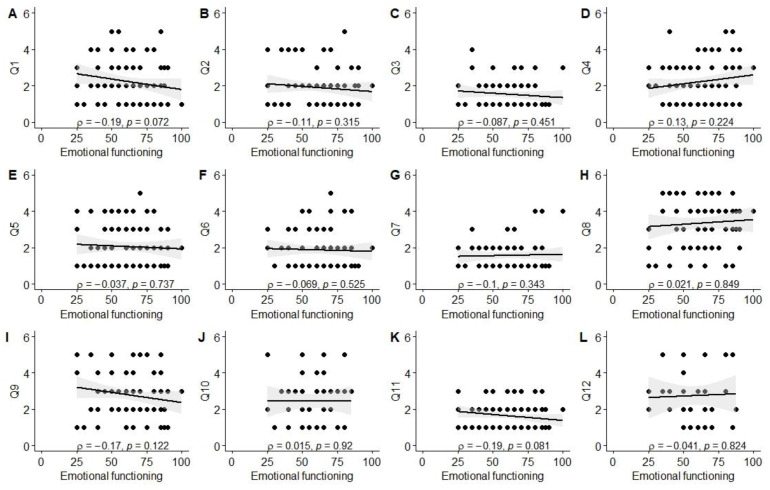
Correlation between emotional functioning score and PREM answers. (**A**) PREM question 1; (**B**) PREM question 2; (**C**) PREM question 3; (**D**) PREM question 4; (**E**) PREM question 5; (**F**) PREM question 6; (**G**) PREM question 7; (**H**) PREM question 8; (**I**) PREM question 9; (**J**) PREM question 10; (**K**) PREM question 11; (**L**) PREM question 12.

**Figure A3:**
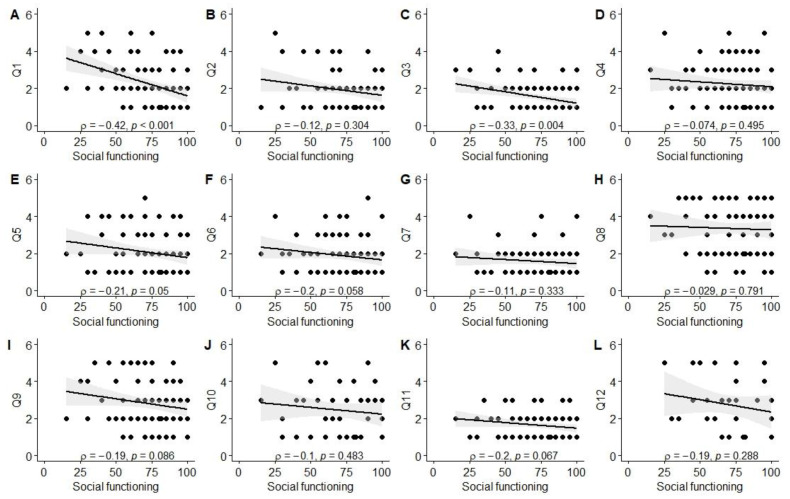
Correlation between social functioning score and PREM answers. (**A**) PREM question 1; (**B**) PREM question 2; (**C**) PREM question 3; (**D**) PREM question 4; (**E**) PREM question 5; (**F**) PREM question 6; (**G**) PREM question 7; (**H**) PREM question 8; (**I**) PREM question 9; (**J**) PREM question 10; (**K**) PREM question 11; (**L**) PREM question 12.

**Figure A4:**
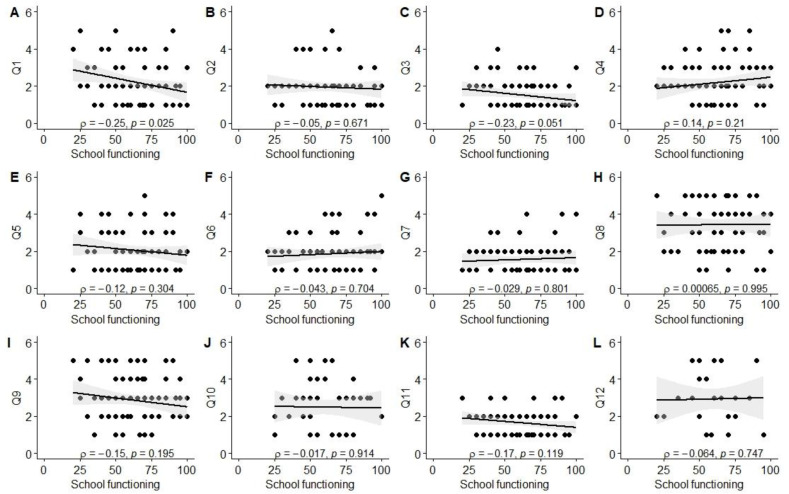
Correlation between school functioning score and PREM answers. (**A**) PREM question 1; (**B**) PREM question 2; (**C**) PREM question 3; (**D**) PREM question 4; (**E**) PREM question 5; (**F**) PREM question 6; (**G**) PREM question 7; (**H**) PREM question 8; (**I**) PREM question 9; (**J**) PREM question 10; (**K**) PREM question 11; (**L**) PREM question 12.

**Figure A5:**
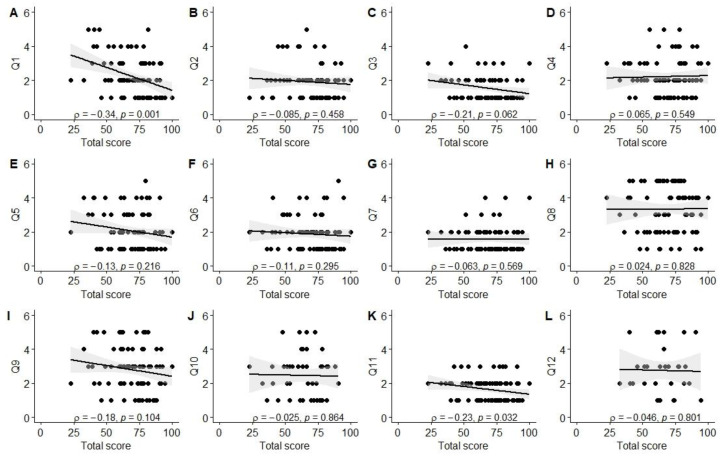
Correlation between total PROM score and PREM answers. (**A**) PREM question 1; (**B**) PREM question 2; (**C**) PREM question 3; (**D**) PREM question 4; (**E**) PREM question 5; (**F**) PREM question 6; (**G**) PREM question 7; (**H**) PREM question 8; (**I**) PREM question 9; (**J**) PREM question 10; (**K**) PREM question 11; (**L**) PREM question 12.

**Figure A6:**
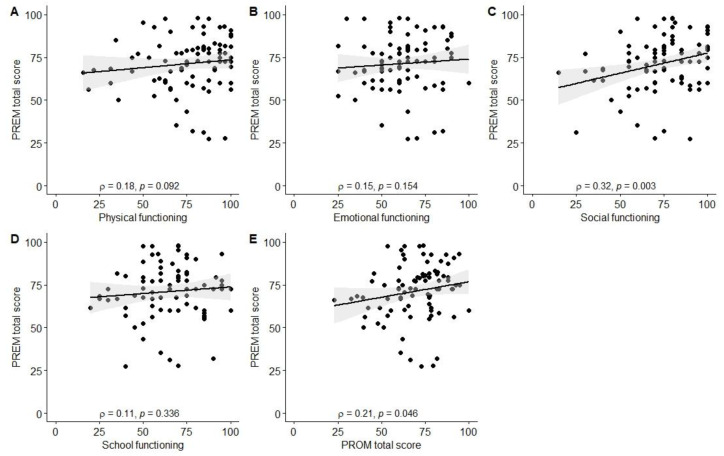
Correlation between total PREM score and PROM dimensions. (**A**) Physical functioning; (**B**) Emotional functioning; (**C**) Social functioning; (**D**) School functioning; (**E**) PROM total schore.

Significant associations were observed between better experiences in the diagnosis establishment process (Q1) and higher scores in physical (ρ = −0.26, *p* = 0.01), social (ρ = −0.42, *p* < 0.001), and school functioning (ρ = −0.25, *p* = 0.03), as well as total PROM score (ρ = −0.34, *p* = 0.001). Better evaluation of the tertiary care center’s environment (Q3; friendliness to children and parents) was associated with higher social functioning scores (ρ = −0.33, *p* = 0.004). Parents reporting better experiences with information provision from healthcare staff (Q11) also reported higher physical functioning (ρ = −0.24, *p* = 0.03) and total PROM scores (ρ = −0.23, *p* = 0.03). Finally, a positive correlation was observed between the total PREM score and the total PROM score (ρ = 0.21, *p* = 0.046), as well as social functioning (ρ = 0.32, *p* = 0.003). The data are also visually summarized in Figure A1, Figure A2, Figure A3, Figure A4, Figure A5 and Figure A6.

## 4. Discussion

In the present study, we aimed to pilot an integrated approach for electronic collection of patient feedback (PROMs and PREMs) in the population of CSHCN within a single specialized tertiary care center. Importantly, we used a custom-designed pilot PREM instrument created with the involvement of all stakeholders and capturing the whole patient journey as opposed to a single encounter. We chose pediatric kidney and hematological diseases as representative conditions associated with long-term healthcare needs and burdens. Our results indicate that such an approach in a real-life setting is feasible and not time consuming, but response rates are relatively low with only half of the parents and even less children responding to the questionnaires. We found the lowest scores for functioning at school and emotional dimensions, with the latter being worse in the hematological diseases group. Although parents rated the experiences in the healthcare system during their child’s patient journey overall favorably, results indicate a lack of being offered psychological support and providing information on social services as the most problematic areas. Finally, we observed several associations between PREMs and PROMs, particularly with the diagnosis establishment process, information provision, and the clinic’s environment.

A recent systematic review analyzing the use of PREMs in pediatric research identified 83 studies (predominantly conducted in the USA) with high heterogeneity in the studied populations, PREM questionnaires employed, and their mode of administration. In the following studies, questionnaires were primarily completed by proxy only, and only 26.5 percent used electronic collection tools. The majority of PREMs were generic and underwent prior validation, with high variability in the domains captured and the number of questions included. This data indicates the lack of a standardized approach and the relatively low use of PREMs in the pediatric setting [17]. This is reiterated by the work of Wray et al., indicating that out of 108 articles reporting use of PROMs/PREMs in routine pediatric hospital care, the absolute majority employed only PROMs, with only seven studies using both PROMs and PREMs [18]. These findings suggest that PREMs remain relatively understudied in the pediatric population, particularly in conjunction with PROMs, and that approaches to using electronic tools for their collection are limited.

A recent qualitative study performed in a single Canadian province analyzing the experience of real-life PROM and PREM users in the pediatric setting has identified five main factors associated with the implementation of PROMs and PREMs in routine practice. These included the characteristics of questionnaires, an individual’s beliefs, the administration of questionnaires, clinical work-flow designs, and incentivization [12]. Similar factors have been identified as potential barriers in the review by Wray et al., including the burden associated with the time needed to complete questionnaires, limited access to resources, and perceived unhelpfulness [18]. Clearly, these factors need to be taken into account when considering a broader implementation of patient-reported measures.

In the aforementioned studies, physicians frequently found that the contents of generic PROMs/PREMs were unsuitable and, thus, of little value for the populations under their care. In our study, we aimed to look at the integrated collection of PROMs and PREMs at the meso/macro-level, i.e., how they reflect healthcare quality at the service provider/national level. Thus, we have chosen to develop a PREM tool that would capture the overall patient journey and give insights into areas of potential improvement at the healthcare service level. Similar approaches with PREMs have already been used in the area of rare diseases [19]. Understanding that such a wide-spread PREM may strongly depend on particular characteristics of the national healthcare system, we have involved all stakeholders (patients, physicians, healthcare managers, and policy makers) in the creation of this pilot custom PREM tool. As for the PROM, we have chosen the PedsQL instrument that is among the most widely used PROMs in the pediatric setting, has both patient and proxy-reporting options, underwent validation in Lithuanian, and is relatively short compared to other instruments [20].

Another reported barrier for successful implementation of PROMs and PREMs in routine practice is considered to be the burden on healthcare staff and patients associated with their administration [12,18]. A systematic review of electronic PROMs collection has revealed benefits of this approach as compared to paper-based collections, including faster completion, reduced costs, and better data quality. On the other hand, the data protection requirements, particularly in the European Union following the General Data Protection Regulations activation, technical issues with the systems, and a lack of computer literacy among older participants may pose challenges to the electronic collection of these measures [13]. In addition, a study with patients in orthopedic practice indicated that automated electronic collection of PROMs was associated with significantly lower response rates when compared to manual collection of PROMs (44% vs. 76%) [21]. These findings are similar to ours: our automated electronic collection approach resulted in an overall response rate of 51%, with even lower results in the children’s group. Low response rates challenge the validity of results, may indicate significant selection bias, and reduce the generalizability of findings. A study involving patients undergoing total hip arthroplasty indicated that when collecting PROMs at two time point the minimum response rate should be 60% [22]. Thus, our findings indicate the need for additional efforts to increase response rates when employing automated electronic collection of patient-reported measures in the pediatric population, particularly targeting children as respondents. On the other hand, we have observed a high rate of fully completed questionnaires (mostly 100%), which may reflect relatively a short completion time (approximately three minutes). The lower response rate in the hematologic malignancies group also points towards a potential differential engagement of patients with different diagnoses. Our study does not allow us to explore the reasons behind this observation, but this may relate to different patient pathways throughout the disease course and should be an object for future studies.

We have observed the lowest emotional and school functioning scores in our population of children with kidney and hematological diseases, which are in line with previous findings in comparable populations [23,24]. This highlights the need for intervention to improve QoL within these domains. However, the use of proxy-reported measures may inaccurately describe the situation as perceived by children themselves. Indeed, previous studies have reported insufficient agreement between child and proxy-reported PedsQL scores [23,25]. We did not find significant differences between the mean scores of all PedsQL dimensions, but the limited sample size of paired child-proxy-reported measures preclude reliable estimation of agreement. However, significant heterogeneity between differences in PROM scores can be observed when looking at individual pair-wise data. On the other hand, the total PREM score differed significantly between parents and children, with the latter reporting better experiences. Whether this represents different perspectives on experiences in the healthcare system (which may also be mediated by differing expectations and prior experiences) or relates to the different contents of both surveys remains unknown and should be an object for future studies.

We also aimed to investigate the self- and parent-reported experiences of CSHCN within the healthcare system throughout their patient journey. The majority of children in our study were diagnosed more than one year ago and had been hospitalized at least once at the time of filling out the survey. This provides sufficient time within the healthcare system to evaluate experiences at different stages of the care process, although the potential confounding effect of heterogeneity in the follow-up period and hospitalization/encounter rate cannot be excluded.

We have observed significant associations between proxy-reported total PREM and total PROM scores, indicating an existing association between patient experiences and outcomes. However, the inherently multifactorial and complex nature of these global scores limits the ability to infer causality or identify specific interactions. Thus, we have looked at the associations between different dimensions of experiences and outcomes. We found that parents’ experiences during the diagnosis establishment process were associated with the physical, social, and school functioning of the child and the total PROM score. Worse experiences in this domain may indicate, among other potential factors, delay in diagnosis (and, consequently, timely management), a lack of coordination between different healthcare sectors, or the complexity/rarity of a disease. Individually or collectively, these factors may mediate the associations with worse proxy-reported QoL indices in children. For example, delay in diagnosis has been associated with poor QoL among different conditions and appears to impact it even after treatment initiation [26,27,28]. Beyond potential anxiety, this has been shown to affect patient-physician interactions and, due to delayed initiation of treatment, also the progression of disease [26]. This points towards the need for clarifying individual components affecting experiences during diagnosis establishment and further investigating whether targeted interventions could translate into improved QoL measures.

We have also observed a significant association between parent perspectives on the clinic’s environment and child- or parent-friendliness and the proxy-reported social functioning of children. Prior research has suggested that the social functioning of children with chronic illnesses appears to be independent of the primary diagnosis but instead associates with individual characteristics of the disease [29]. Moreover, those with chronic diseases are typically more restricted from routine social activities by requiring more time to be spent in the hospital environment. Hospital environments have been recognized as an important contributor to psychosocial functioning in both children and adult populations [30,31]. In pediatric oncology, various interventions, including camps, support groups, and organized activities in the hospital, have been studied in order to improve the social outcomes of children [32]. Our data also reiterates and highlights the importance of staff-patient interactions and the hospital environment as potential targets for interventions aiming to improve the social functioning of CSHCN.

Finally, we have found associations between information provision from the healthcare staff and proxy-reported physical functioning of the child, as well as the total PROM score. A review of studies involving cancer survivors has revealed a strong link between information provision and health related QoL, depression, and anxiety. However, interventions to improve information provision mostly failed to show improvement in QoL dimensions [33], likely indicating a cumulative effect of different dimensions of healthcare quality. The associations between satisfaction with information provision and different dimensions have been reported in different patient populations, with some suggestions of a decrease over time [34,35,36]. Nevertheless, our study further highlights the importance of sufficient information provision on patient-reported outcomes and the need to address this issue during the holistic healthcare delivery process in the CSHCN population.

Our study is subject to several limitations. First, due to the requirement to collect email addresses we had to obtain informed consent, so patients who refused to participate limited the generalizability of our findings. We were unable to collect information on the reasons for non-response, which further increases the risk of selection bias. As the primary aim of our study was to pilot our approach within the CSHCN population by using the example of kidney and hematological diseases at the healthcare provider level, we used aggregated data for analysis of these groups, and looking at specific diagnoses was beyond our scope. The remote filling of questionnaires could not be monitored, and thus, whether children completed questionnaires without their parents being present cannot be confirmed. However, both parents and children were instructed that children should complete questionnaires on their own. Relatively low sample sizes and low response rates for child-reported questionnaires limited our ability to perform inferential analysis in this subgroup. Due to the cross-sectional nature of our pilot study, we were unable to track these patient-reported measures longitudinally and assess patients’ and physicians’ perspectives on their value. The latter approach may have allowed us to better evaluate associations between PREMs and PROMs and to better assess the strength of this relationship. In our study, we also were unable to evaluate the impact of our integrated patient feedback collection on healthcare quality improvement, which may be challenged by financial and human resource constraints. Finally, for this pilot study, we used a custom-created PREM instrument that may limit its generalizability to other healthcare systems and a generic PROM instrument that may lack specificity in certain dimensions for the studied population.

In conclusion, we have piloted an integrated approach for automated electronic collection of PROMs and PREMs in the CSHCN population, aiming to explore its feasibility and potential associations of patient experience monitoring throughout the whole patient journey with subjective health outcomes. Our data indicates that such an approach is feasible, but efforts are required (e.g., small incentives for children) to increase response rates and improve the validity of results. Our data allows us to identify priority outcomes to be targeted in pediatric kidney and hematology patient populations that primarily include emotional and school functioning. In addition, information provision on social services and offering psychological support appear to be the most problematic areas among parents, while children indicate a lack of help to continue everyday activities. Importantly, the association between certain dimensions of PREMs and PROMs indicate the potential of targeted interventions to improve healthcare service delivery and patient-reported outcomes that could be tested in appropriately designed studies. Further exploratory and interventional studies, particularly targeting the specification of individual components of experiences during the diagnosis establishment process, the hospital’s environment and staff friendliness, and information provision, should be considered. Finally, more studies aiming to capture patient journeys and outcomes using population-specific PROM and PREM tools in larger real-life longitudinal studies that include assessment of patient and physician perspectives are needed to clarify the value of such an approach.

## Figures and Tables

**Table 1:** Number of questionnaires sent and respective response rates.

	Number of Questionnaires and Response Rates		
**Overall**	**Total** (*n* = 450)	**PROM** (*n* = 219)	**PREM** (*n* = 231)
	230 (51.1%)	109 (49.8%)	121 (52.4%)
**Parents**	**Total** (*n* = 364)	**PROM** (*n* = 176)	**PREM** (*n* = 188)
	198 (54.4%)	94 (53.4%)	104 (55.3%)
**Children**	**Total** (*n* = 86)	**PROM** (*n* = 43)	**PREM** (*n* = 43)
	32 (37.2%)	15 (34.9%)	17 (39.5%)

**Table 2:** Time since diagnosis and time since last hospitalization by disease group.

Characteristic	Overall (*n* = 104)	Kidney Disease (*n* = 62)	Hematological Disease (*n* = 42)	*p*-Value
**How long ago was your child’s disease diagnosed?**				<0.001 ^1^
Less than 1 year ago	18 (17.3%)	9 (14.5%)	9 (21.4%)	
1–2 years ago	33 (31.7%)	10 (16.1%)	23 (54.8%)	
3 or more years ago	53 (51.0%)	43 (69.4%)	10 (23.8%)	
**When was the last time your child was admitted to the hospital?**				0.002 ^2^
My child has not been admitted to the hospital	5 (4.8%)	4 (6.5%)	1 (2.4%)	
Less than 1 year ago	49 (47.1%)	20 (32.3%)	29 (69.0%)	
1–2 years ago	31 (29.8%)	22 (35.5%)	9 (21.4%)	
3 or more years ago	19 (18.3%)	16 (25.8%)	3 (7.1%)	

^1^ Pearson’s Chi-squared test; ^2^ Fisher’s exact test.

## Data Availability

The data presented in this study are available from the authors on request.

## Appendix A

**Table A1:** PREM questionnaire for children (translated to English from Lithuanian).

Q1	**Doctors and nurses at my outpatient clinic * are friendly.** Family physician/pediatrician, nurse and other staff members at my outpatient clinic are nice and try to make me feel good.
	I do not attend a doctor at the outpatient clinic Strongly agree Agree Neither agree nor disagree Disagree Strongly disagree
Q2	**Attending the outpatient clinic does not scare me.** When I visit my family physician/pediatrician, the clinic environment does not frighten me and I feel comfortable and safe.
	I do not attend a doctor at the outpatient clinic Strongly agree Agree Neither agree nor disagree Disagree Strongly disagree
Q3	**Doctors, nurses, and receptionists at the Santaros ** outpatient clinic/daycare clinic are friendly and helpful.** When I see a doctor in the hospital or day hospital, I feel good. They take care of me and explain about upcoming procedures and tests.
	I do not attend Santaros outpatient clinic/daycare clinic Strongly agree Agree Neither agree nor disagree Disagree Strongly disagree
Q4	**I was explained about my illness in an easy to understand way.** I was explained about my condition, results of my tests and how I will be treated.
	Strongly agree Agree Neither agree nor disagree Disagree Strongly disagree
Q5	**During my admission(s) in the hospital, I felt that I was cared for.** When I was admitted to the hospital, doctors, nurses and other staff members were friendly, tried to help me and made me feel good.
	I have not been admitted to the hospital Strongly agree Agree Neither agree nor disagree Disagree Strongly disagree
Q6	**I received help to continue my normal everyday activities.** I was given an opportunity to continue my education while being in the hospital or at home. I received help to continue my education in a different way in case this was required.
	Strongly agree Agree Neither agree nor disagree Disagree Strongly disagree
Q7	**I felt that during my rehabilitation I received help to become stronger.** I generally liked my rehabilitation process. I felt that there was an effort made to make me feel good, to restore my strength and my mood.

* In the original language (Lithuanian) this referrers to primary care outpatient clinics. ** Santaros clinics is the tertiary care center where patients were managed for their primary disease.

**Table A2:** PREM questionnaire for parents (translated to English from Lithuanian).

**How long ago was your child’s disease diagnosed?**	
	Less than 1 year ago 1–2 years ago 3 or more years ago
**When was the last time your child was admitted to the hospital?**	
	My child has not been admitted to the hospital Less than 1 year ago 1–2 years ago 3 or more years ago
Q1	**The process from the onset of symptoms to diagnosis establishment was smooth.** Since the onset of the first symptoms, we had no difficulty getting to our doctor, other specialists and specialized tests.
	Strongly agree Agree Neither agree nor disagree Disagree Strongly disagree
Q2	**The environment of the primary care outpatient * clinic is child- and parent-friendly**. The staff at the clinic is friendly, tries to understand our needs and is always ready to help. We did not experience negative attitudes or inappropriate behavior and the clinic environment is designed to make the child feel good.
	My child does not attend primary care outpatient clinic Strongly agree Agree Neither agree nor disagree Disagree Strongly disagree
Q3	**The environment of the Santaros ** outpatient clinic is child- and parent-friendly.** The staff at the clinic is friendly, tries to understand our needs and is always ready to help. We did not experience negative attitudes or inappropriate behavior and the clinic environment is designed to make the child feel good.
	My child does not attend Santaros outpatient clinic Strongly agree Agree Neither agree nor disagree Disagree Strongly disagree
Q4	**Family physician/pediatrician is involved in the treatment of my child’s condition.** Family physician/pediatrician and nurses are knowledgeable about my child’s condition, can provide requested information and try to help us.
	Strongly agree Agree Neither agree nor disagree Disagree Strongly disagree
Q5	**I have not encountered any technical problems in healthcare service provision**. I did not encounter issues during referral, electronic prescription or drug reimbursement procedures for my child.
	Strongly agree Agree Neither agree nor disagree Disagree Strongly disagree
Q6	**I have received sufficient information about my child’s condition**. Following the diagnosis of my child’s disease, I received sufficient information about my child’s condition and its management.
	Strongly agree Agree Neither agree nor disagree Disagree Strongly disagree
Q7	**The environment of the hospital’s in-patient departments is child- and parent-friendly.** The staff at the in-patient departments is friendly, tries to understand our needs and is always ready to help. We did not experience negative attitudes or inappropriate behavior and the clinic environment is designed to make the child feel good.
	My child has not been admitted to the hospital Strongly agree Agree Neither agree nor disagree Disagree Strongly disagree
Q8	**We have been offered professional psychological help**. We were provided professional psychological support, including in-patient treatment.
	Strongly agree Agree Neither agree nor disagree Disagree Strongly disagree
Q9	**I received sufficient information about available social services**. After the diagnosis and during treatment, I received sufficient information about all additional social services, sick-leave process, disability procedures and other assistance options.
	Strongly agree Agree Neither agree nor disagree Disagree Strongly disagree
Q10	**Options were provided to continue my child’s education.** During the stays in hospital, as well as during the outpatient treatment (if school attendance was not possible), my child was given the opportunity to study in order to keep up with peers.
	There was no such need Strongly agree Agree Neither agree nor disagree Disagree Strongly disagree
Q11	**I received sufficient information about the treatment of my child’s condition at home**. Clear information about further management of my child was provided and included recommendations for everyday living at home, medication administration, as well as contact information.
	Strongly agree Agree Neither agree nor disagree Disagree Strongly disagree
Q12	**During rehabilitation my child received the best possible care to help him/her recover and get stronger.**
	My child did not require rehabilitation Strongly agree Agree Neither agree nor disagree Disagree Strongly disagree

* In the original language (Lithuanian) this referrers to primary care outpatient clinic. ** Santaros clinics is the tertiary care center where patients were managed for their primary disease.

**Table A3:** Psychometric evaluation of the questionnaires.

	Cronbach Alpha	95% Confidence Interval	
		Upper	Lower
**PROM (parents)**			
Age group (years)			
0–1	-	-	-
2–4	0.631	0.531	0.719
5–7	0.916	0.902	0.929
8–12	0.909	0.900	0.917
13–18	0.953	0.946	0.959
**PREM (parents)**			
All	0.856	0.829	0.881
Disease group			
Kidney	0.792	0.739	0.839
Hematological	0.902	0.873	0.927
Age group (years)			
0–1	-	-	-
2–4	0.554	0.304	0.742
5–7	0.94	0.906	0.965
8–12	0.885	0.854	0.912
13–18	0.782	0.679	0.863
Child’s gender			
Girls	0.928	0.905	0.948
Boys	0.77	0.713	0.82

**Table A4:** Response rate by questionnaire type and patient group.

**PROM Questionnaires**			
**Overall**	**Total** (*n* = 450)	**Kidney disease** (*n* = 122)	**Hematological disease** (*n* = 97)
	109 (49.8%)	66 (54.1%)	43 (44.3%)
**Parents**	**Total** (*n* = 176)	**Kidney disease** (*n* = 91)	**Hematological disease** (*n* = 85)
	94 (53.4%)	54 (59.3%)	40 (47.1%)
**Children**	**Total** (*n* = 43)	**Kidney disease** (*n* = 31)	**Hematological disease** (*n* = 12)
	15 (34.9%)	12 (38.7%)	3 (25.0%)
**PROM questionnaires**			
**Overall response rate**	**Total** (*n* = 231)	**Kidney disease** (*n* = 128)	**Hematological disease** (*n* = 103)
	121 (52.4%)	74 (57.8%)	47 (45.6%)
**Parents**	**Total** (*n* = 188)	**Kidney disease** (*n* = 97)	**Hematological disease** (*n* = 91)
	104 (55.3%)	62 (63.9%)	42 (46.2%)
**Children**	**Total** (*n* = 43)	**Kidney disease** (*n* = 31)	**Hematological disease** (*n* = 12)
	17 (39.5%)	12 (38.7%)	5 (41.7%)

**Table A5:** Completion times of parent questionnaires by patient subgroup, age group and gender.

	PROM Parents									
Completion Time		Disease Group		Age Group (Years)					Child’s Gender	
	**Total** (*n* = 94)	**Kidney disease** (*n* = 54)	**Hematological disease** (*n* = 40)	**0–1** (*n* = 0)	**2–4** (*n* = 12)	**5–7** (*n* = 17)	**8–12** (*n* = 40)	**13–18** (*n* = 25)	**Girls** (*n* = 34)	**Boys** (*n* = 60)
**Median (IQR)**	3.1 (2.3–4.0)	3.1 (2.3–3.8)	3.2 (2.3–4.4)	-	3.5 (2.3–4.1)	2.8 (2.2–3.7)	3.0 (2.4–3.8)	3.4 (2.2–4.5)	3.4 (2.7–5.0)	2.9 (2.2–3.8)
**Min–max**	1.3–17.8	1.3–12.8	1.3–17.8	-	1.7–7.4	1.3–10.6	1.7–11.6	1.3–17.8	1.5–17.8	1.3–12.8
	**PREM parents**									
		**Disease group**		**Age group (years)**					**Child’s gender**	
	**Total** (*n* = 104)	**Kidney disease** (*n* = 62)	**Hematological disease** (*n* = 42)	**0–1** (*n* = 7)	**2–4** (*n* = 13)	**5–7** (*n* = 16)	**8–12** (*n* = 42)	**13–18** (*n* = 26)	**Girls** (*n* = 42)	**Boys** (*n* = 62)
**Median (IQR)**	3.2 (2.4–4.1)	3.3 (2.4–4.1)	3.0 (2.5–4.2)	3.2 (2.7–4.0)	2.8 (2.5–3.2)	2.8 (2.3–3.9)	3.3 (2.6–4.1)	3.1 (2.3–4.3)	3.3 (2.5–4.4)	3.1 (2.4- 3.9)
**Min–max**	1.5–14.7	1.5–14.7	1.6–7.8	2.5–4.7	2.4–6.4	1.6–6.8	1.9–14.7	1.5–8.0	1.5–14.7	1.5–10.6

**Table A6:** Completion times of children questionnaires by patient subgroup, age group and gender.

	**PROM Children**						
**Completion Time**		**Disease Group**				**Child’s Gender**	
	**Total** (*n* = 15)	**Kidney disease** (*n* = 12)	**Hematological disease** (*n* = 3)	**8–12** (*n* = 6)	**13–18** (*n* = 9)	**Girls** (*n* = 8)	**Boys** (*n* = 7)
**Median (IQR)**	2.4 (1.6–2.9)	2.2 (1.6–2.9)	2.6 (2.1–2.7)	2.9 (2.2–3.0)	2.2 (1.5–2.8)	2.7 (2.0–2.9)	2.0 (1.4–2.7)
**Min–max**	1.4–7.0	1.4–7.0	1.6–2.8	1.7–6.3	1.4–7.0	1.5–7.0	1.4–6.3
	**PREM children**						
**Completion time**		**Disease group**		**Age group (years)**		**Child’s gender**	
	**Total** (*n* = 17)	**Kidney disease** (*n* = 12)	**Hematological disease** (*n* = 5)	**8–12** (*n* = 5)	**13–18** (*n* = 12)	**Girls** (*n* = 8)	**Boys** (*n* = 9)
**Median (IQR)**	1.5 (1.0–2.5)	1.3 (1.0–2.5)	2.0 (1.5–2.4)	2.6 (1.4–2.9)	1.3 (1.0–2.2)	1.4 (1.0–2.1)	2.2 (1.2–2.6)
**Min-max**	0.7–3.1	0.7–3.1	1.0–2.9	1.3–3.1	0.7–2.9	0.7–2.9	0.9–3.1
